# Intestinal enteroids recapitulate the effects of short-chain fatty acids on the intestinal epithelium

**DOI:** 10.1371/journal.pone.0230231

**Published:** 2020-04-02

**Authors:** Sarah C. Pearce, Gregory J. Weber, Dana M. van Sambeek, Jason W. Soares, Kenneth Racicot, David T. Breault

**Affiliations:** 1 Performance Nutrition Team, Combat Feeding Directorate, Combat Capabilities Development Command Soldier Center, Natick, Massachusetts, United States of America; 2 Biological Sciences & Technology Team, Soldier Performance Optimization Directorate, Combat Capabilities Development Command Soldier Center, Natick, Massachusetts, United States of America; 3 Division of Endocrinology, Boston Children’s Hospital, Harvard Medical School, Boston, Massachusetts, United States of America; 4 Harvard Stem Cell Institute, Cambridge, Massachusetts, United States of America; 5 Department of Pediatrics, Harvard Medical School, Boston, Massachusetts, United States of America; Southern Illinois University School of Medicine, UNITED STATES

## Abstract

Enteroids are cultured primary intestinal epithelial cells that recapitulate epithelial lineage development allowing for a more complex and physiologically relevant model for scientific study. The large presence of intestinal stem cells (ISC) in these enteroids allows for the study of metabolite effects on cellular processes and resulting progeny cells. Short-chain fatty acids (SCFA) such as butyrate (BUT) are bacterial metabolites produced in the gastrointestinal tract that are considered to be beneficial to host cells. Therefore, the objective was to study the effects of SCFAs on biomarkers of ISC activity, differentiation, barrier function and epithelial defense in the intestine using mouse and human enteroid models. Enteroids were treated with two concentrations of acetate (ACET), propionate (PROP), or BUT for 24 h. Enteroids treated with BUT or PROP showed a decrease in proliferation via EdU uptake relative to the controls in both mouse and human models. Gene expression of *Lgr5* was shown to decrease with BUT and PROP treatments, but increased with ACET. As a result of BUT and PROP treatments, there was an increase in differentiation markers for enterocyte, Paneth, goblet, and enteroendocrine cells. Gene expression of antimicrobial proteins *Reg3β*, *Reg3γ*, and *Defb1* were stimulated by BUT and PROP, but not by ACET which had a greater effect on expression of tight junction genes *Cldn3* and *Ocln* in 3D enteroids. Similar results were obtained with human enteroids treated with 10 mM SCFAs and grown in either 3D or Transwell^™^ model cultures, although tight junctions were influenced by BUT and PROP, but not ACET in monolayer format. Furthermore, BUT and PROP treatments increased transepithelial electrical resistance after 24 h compared to ACET or control. Overall, individual SCFAs are potent stimulators of cellular gene expression, however, PROP and especially BUT show great efficacy for driving cell differentiation and gene expression.

## Introduction

The host-microbiome interaction is critically important for maintaining homeostasis within the gastrointestinal tract. The mammalian intestine is responsible for nutrient digestion and absorption, as well as ion and water transport. However, the intestine cannot digest certain types of nutrients such as dietary fiber, and therefore relies on the gut microbiota to ferment or bio-transform these compounds into usable fuel sources [1]. Specifically, the gut microbiota plays a large role in maintaining gut health in part by metabolizing nutrients to create short-chain fatty acids (SCFAs) and other beneficial metabolites such as phenolic metabolites utilized by the host [2]. In addition, bacteria normally found in the mammalian gut synthesize vitamins including Vitamin K and Vitamin B12 [3]. Bacterial-derived SCFAs including acetate (ACET), butyrate (BUT) and propionate (PROP) are largely derived from saccharolytic fermentation by anaerobic bacteria in the intestine. Short chain fatty acids in the terminal ileum have been found at a concentration of ~13 mM while the large intestine contains ~100 mM.

The epithelial barrier is the main host defense mechanism against bacteria, viruses, fungi, and yeast. Epithelial cells produce mucin as a first layer defense and secrete antimicrobial peptides, chemokines, and cytokines [4]. As bacterial by-products, SCFAs (largely BUT) are in close contact with epithelial cells and are taken up where they are then utilized as an energy source by absorptive cells. Short chain fatty acids are also known to exhibit effects on cellular proliferation and immune response [5]. Previous literature has shown that BUT decreases cellular proliferation and increases antimicrobial peptide expression, cytokine/chemokine production, as well as tight junction proteins [6, 7]. BUT has been the most widely studied SCFA and has been reported to have specific health benefits [8]. However, negative effects of BUT have been observed during dysbiosis, which is characterized by a compositional shift from obligate anaerobes to facultative anaerobes within the gut microbiome community. Over production of BUT or epithelial damage can lead to aberrant BUT signaling through the histone deacetylase pathway, causing decreased stem cell proliferation, which is critical for intestinal cell turnover [9, 10]. The small intestine (SI) is a difficult environment for bacteria due to short transit times combined with presence of bile and digestive enzymes. Despite this, unique bacterial communities reside throughout the length of the small intestine, although the SI microbiome is far less complex than that found in the colon. Interestingly, concentrations of ACET and BUT in the small intestine have been reported to be similar to that observed in fecal samples (~60 mM and ~12 mM), while PROP is 3–5 times lower at ~3 mM [11].

Physiologically relevant models to examine effects of SCFAs and other nutrients/metabolites on epithelial function have been limited until recently, with new advances in primary intestinal enteroid models that recapitulate *in vivo* intestinal cell types and function [12–14]. More recent advancements have included optimization of human intestinal enteroid monolayers that create a polarized model to study intestinal barrier properties and nutrition even more effectively [15, 16]. In addition, these models provide an ability to harvest tissue from human patients, therefore reducing translatability issues. A majority of *in vitro* SCFA research has focused on transformed cell lines, which has provided extensive information on SCFA mechanisms. By comparison, less is known with regard to the effects of SCFA microbial metabolites on a multicellular system such as enteroids but recent studies have begun to demonstrate the potential of this model to study microbial metabolites [17–20]. In addition, the largest body of evidence regarding SCFA effects on the host focuses on BUT, while less is known about the effects of two of the other main SCFAs, ACET and PROP. Therefore, our objectives were to determine how individual SCFAs affect intestinal epithelial barrier function, epithelial inflammation, and cell fate using complex physiologically relevant 2D and 3D intestinal enteroid models from mouse and human. We hypothesized that SCFAs would exert similar effects in enteroids to those found *in vivo*.

## Materials and methods

### Enteroids

De-identified endoscopic tissue biopsies were collected from grossly unaffected (macroscopically normal) areas of the duodenum in 10–14 year old patients undergoing endoscopy for gastrointestinal complaints. Informed consent and developmentally-appropriate assent were obtained at Boston Children’s Hospital from the donors’ guardian and the donor, respectively. All methods were carried out in accordance with the Institutional Review Board of Boston Children’s Hospital (Protocol number IRB-P00000529) approval. Tissue was digested in 2 mg ml^−1^ collagenase I for 40 min at 37°C followed by mechanical dissociation, and isolated crypts were resuspended in growth factor-reduced Matrigel and obtained as frozen enteroids at low passage number. Duodenal enteroids from wild-type C57BL/6 mice were also obtained under IACUC approval from the source laboratory (Harvard/Boston Children’s Hospital).

### 3D cultures

Frozen enteroids were thawed and immediately transferred to a 15 mL conical tube with 5 mL of complete media without growth factors (CMGF-)containing Advanced DMEM/F12, 0.2 mM Glutamax, and 10 mM HEPES. Cells were then gently spun down at 300 x g for 10 min and supernatant removed. Enteroid pellets were resuspended in growth-factor reduced Matrigel (#356231, Corning, Corning, NY). Aliquots (40 μL) containing ~100 enteroids were plated in individual wells of a 24-well tissue culture treated plate and incubated at 37°C for 10 min before adding 0.5 mL of media (Mouse or Human Intesticult^™^ Stem Cell Technologies, Cambridge, MA). Media was replaced every two days, and enteroids were passaged every 5–7 d by incubation in Gentle Cell Dissociation Reagent (Stem Cell Technologies, Cambridge, MA) at 4°C with shaking for 40 min. Well contents were scraped and triturated with a P200 pipette tip 30–50 times to break apart enteroids, collected in a 15 mL conical tube with 1:1 addition of CMGF- media and centrifuged at 300 x g for 10 min. Cell pellets were resuspended in Matrigel to achieve a similar density each time. Experiments were conducted on enteroids between passages 10–15.

### 2D monolayer cultures

Monolayer protocols were adapted from previous publications [13]. Enteroids were initially cultured in matrigel for 2–3 passages prior to plating on monolayers. To form monolayers, Transwell^™^ inserts (24-well inserts, 0.33 cm^2^ surface area, 0.4 μm pore polyester membrane; Corning, Corning, NY) were coated with human collagen IV solution (final concentration of 10 μg/cm^2^) and incubated overnight at 4°C. Human collagen IV (Millipore Sigma, Burlington, MA) was purchased as a liquid in 0.5 M acetic acid, then diluted using sterile water. Prior to plating, any remaining collagen was removed from wells and washed 2X with Advanced DMEM/F12. Fragments for monolayer plating were obtained using the passaging protocol above. Approximately 50 enteroid fragments were obtained per 100 μL Intesticult^™^ media, then added to the filter and allowed to settle at 37°C. 600 μL Intesticult^™^ media was also added to the basolateral side. Media was changed every 2 d and monolayer development was tracked using transepithelial electrical resistance (TER) measured by the EVOM2 epithelial voltohmmeter (World Precision Instruments, Sarasota, FL).

### Short-chain fatty acid treatment

Short-chain fatty acids ACET, BUT, and PROP (Sigma Aldrich, St. Louis, MO) in salt form diluted in purified water were used to treat enteroids at physiologically relevant small intestinal concentrations for mouse (1 and 5 mM) and human (1 and 10 mM) for 24 h based on previous enteroid and non-enteroid cell literature [18].

### RNA extraction & mRNA analysis

Total RNA was extracted from intestinal enteroids using a commercially available kit (RNeasy Micro, Qiagen). cDNA was synthesized using the Maxima First Strand cDNA Synthesis Kit (ThermoFisher Scientific, Waltham MA). Real-time polymerase chain reaction using a BioRad CFX Connect (Hercules, CA) was used to analyze mRNA expression using Maxima SYBR green (ThermoFisher Scientific, Waltham MA). Data were analyzed using the Delta-Delta Ct (2^–ΔΔCt^) method and presented as relative fold gene expression. Treatments were all made relative to controls, which were set to equal one. All samples were standardized to β–actin expression and β–actin values were determined to be statistically similar between treatment groups (S1 Fig). Primer sequences (Integrated DNA Technologies, Coralville, IA) are listed in Additional file 1: S1a and S1b Table.

### EdU labeling

5-Ethynyl-2’deoxyuridine (EdU) labeling was carried out using the Click-iT EdU Alexa Fluor 488 Imaging Kit (Life Technologies). 3D enteroids were cultured on chamber slides (Nunc Lab-Tek II; ThermoFisher Scientific, Waltham MA) while 2D monolayers were grown on Transwell^™^ inserts prior to staining. Following experiments, enteroids were incubated with 10 mM EdU for 3 h and fixed with 10% Neutral Buffered Formalin (NBF) at room temperature for 10 min. Permeabilization and the Click-iT reaction were carried out as described in the kit protocol. Chamber slides or Transwell^™^ membranes were mounted with coverslips containing ProLong^™^ Gold Antifade Mounting Media (ThermoFisher Scientific, Waltham, MA) and imaged using a Zeiss LSM 710 confocal microscope with a 40X objective (Carl Zeiss, Oberkochen, Germany). ImageJ (National Institutes of Health, https://imagej.nih.gov/ij/) was utilized to process and quantify number of labeled cells versus total number of nuclei. All treatments were made relative to controls.

### Immunofluorescence staining

3D enteroids were cultured on chamber slides (Nunc Lab-Tek II^™^; ThermoFisher Scientific, Waltham MA) while 2D monolayers were grown on Transwell^™^ inserts prior to staining. Enteroids in both conditions were fixed in 10% NBF at room temperature for 20 min, followed by permeabilization in Triton x 100 for 20 min [21]. 3D murine enteroids were then stained with F-actin marker, ActinRed^™^ 555 ReadyProbes Reagent (Invitrogen, ThermoFisher Scientific, Waltham, MA), while 2D enteroids were stained with tight junction proteins claudin-3 (CLDN3) and zonula occludens-1 (ZO1; Abcam, Cambridge, MA). Samples were mounted with coverslips containing ProLong^™^ Gold Antifade Mounting medium (ThermoFisher Scientific, Waltham, MA) and imaged using a confocal microscope.

### Histological staining

Mucin production was analyzed using a commercially available stain (Alcian Blue pH 2.5 Stain Solution; Richard-Allan Scientific, ThermoFisher Scientific, Waltham, MA). Briefly, cells grown on Transwell^™^ inserts were fixed in 10% Neutral Buffered Formalin (NBF) at room temperature for 20 min and then were placed in Alcian blue for 30 min at room temperature. Cells were also counterstained with nuclear marker hematoxylin for 5 min. Following staining, inserts were washed several times with deionized water and then mounted with coverslips containing ProLong^™^ Gold Antifade Mounting Media (ThermoFisher Scientific, Waltham, MA) and imaged using a confocal microscope.

### Secreted protein analyses

Protein supernatant extracted from 2D enteroids using RIPA buffer as well as basolateral media from enteroids cultured in 2D were analyzed for goblet cell marker Trefoil Factor 3 (TFF3) using a commercially available ELISA kit, per manufacturer’s instructions (R&D Systems, Minneapolis, MN). Additional proteins including Interleukin-4 (IL-4), Interleukin-8 (IL-8), Interleukin-18 (IL-18), soluble RANK ligand (sRANKL), ghrelin and Peptide YY (PYY) were analyzed using a custom ProcartaPlex Immunoassay Kit using antibody-based magnetic beads (ThermoFisher Scientific, Waltham MA). Samples were read and analyzed on a Luminex MAGPIX multiplexing system (Luminex, Northbrook, IL). Samples were normalized using protein expression as measured by a commercially available Bicinchoninic acid assay (BCA; ThermoFisher Scientific, Waltham MA).

### Western blotting

Whole cell protein from 2D enteroids was extracted with a commercial RIPA buffer with protease and phosphatase inhibitors. Cell lysates were loaded with 4X Laemmli Sample Buffer (Bio Rad, Hercules, CA) and headed at 95°C for 6 minutes. Samples were then separated by sodium dodecyl sulfate (4–20%) polyacrylamide gel electrophoresis (SDS-PAGE). Gels were run under reducing conditions and transferred to Polyvinylidene difluoride (PVDF) membranes. Membranes were blocked for 1 h in 5% non-fat dry milk (NFDM) in Tris-buffered saline with tween (TBST; 1X TBS, 0.1% Tween-20). Membranes were then incubated in primary antibody with 5% NFDM in TBST overnight at 4°C. After incubation in primary antibody (Zonula occludens-1, Claudin-3, Occludin, Chromogranin-A, Trefoil Factor-3, and β-Actin), membranes were incubated in horseradish peroxidase (HRP)-conjugated secondary antibody for 1 h at room temperature. For detection, Supersignal West Pico Chemiluminescent Substrate was utilized (Thermofisher Scientific, Waltham, MA). Membranes were imaged using a Syngene ChemiGenius (Syngene, Fredrick, MD). Band densities were quantified using ImageJ (NIH). Bands were standardized to the density of β-Actin and represented as a ratio of each protein to β-Actin.

### Statistical analysis

Experiments were analyzed using JMP SAS (SAS Institute, Cary, NC). Individual wells were used as the experimental unit and biological replicates were also analyzed to confirm results. Data were analyzed with a one-way ANOVA with a Tukey-Kramer adjustment to compare means across treatments. Data are presented as least square means ± standard error of the mean. Data was considered significant at *P* < 0.05. Superscripts (a,b,c,d) indicate statistical differences between treatments and are compared to controls which are set to 1.0.

## Results

### 3D murine duodenal enteroid morphology changes due to SCFA treatment

**Fig 1:**
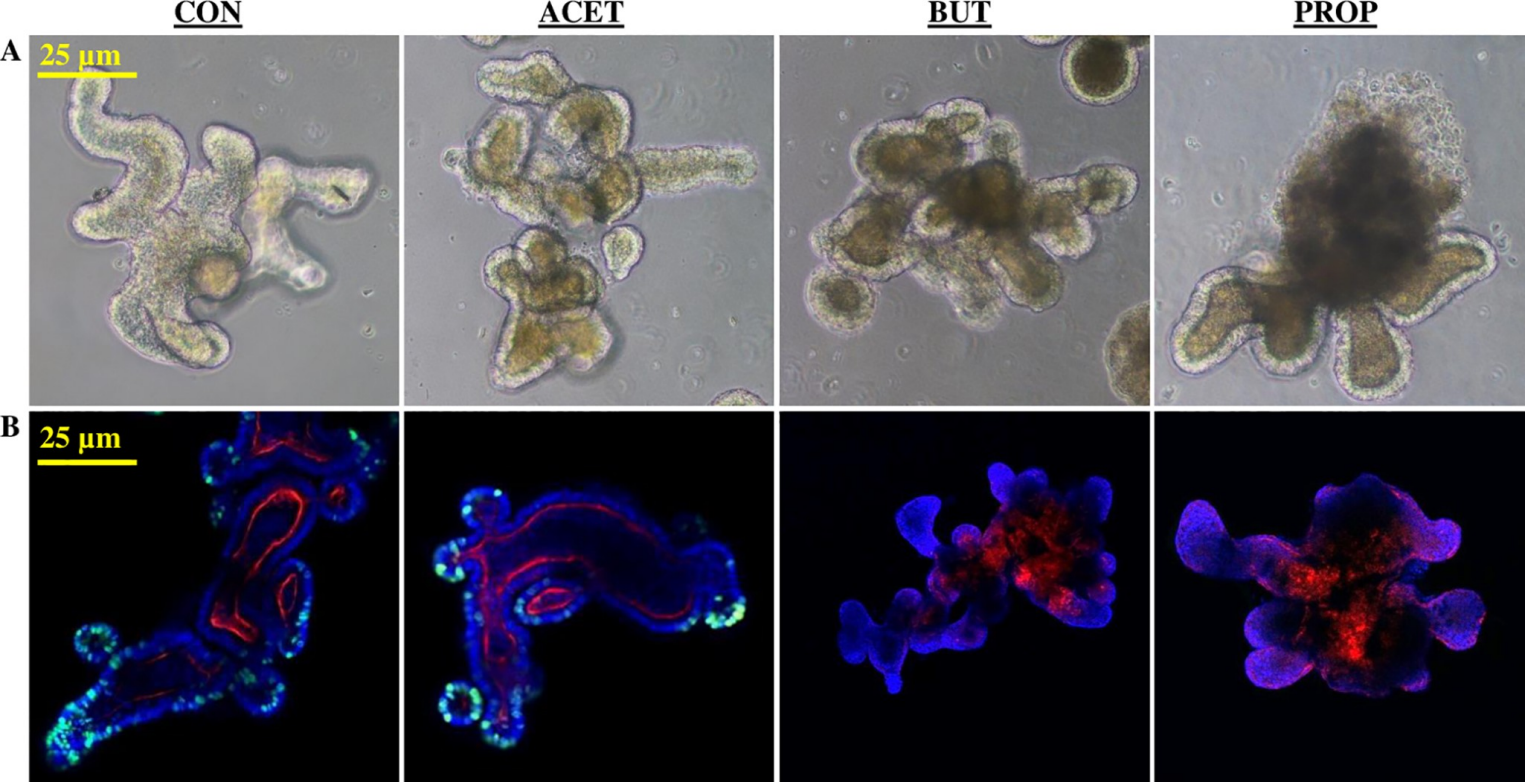
(A) Effect of 24 h treatment with 5 mM acetate (ACET), butyrate (BUT), or propionate (PROP) on morphology of 3D murine duodenal enteroids as well as (B) EdU cellular proliferation marker (green), and F-actin distribution (red) compared to controls (CON). Nuclei are stained in with DAPI (blue) and images are representative of several fields of view at 20X magnification.

Murine enteroids treated with 5 mM SCFAs exhibited altered morphology as observed by bright field microscopy (Fig 1). Treatment with SCFA, especially BUT and PROP caused enteroids to acquire defined luminal structures with a large degree of cell “browning” or cell exfoliation within the lumens (Fig 1a). Cell proliferation, measured by EdU incorporation showed BUT and PROP treatment caused a complete cessation in cell proliferation and changes in F-actin, a structural marker, while ACET treatment appeared similar to that of the control (CON) enteroids (Fig 1b).

### Gene expression in 3D murine duodenal enteroids

**Fig 2:**
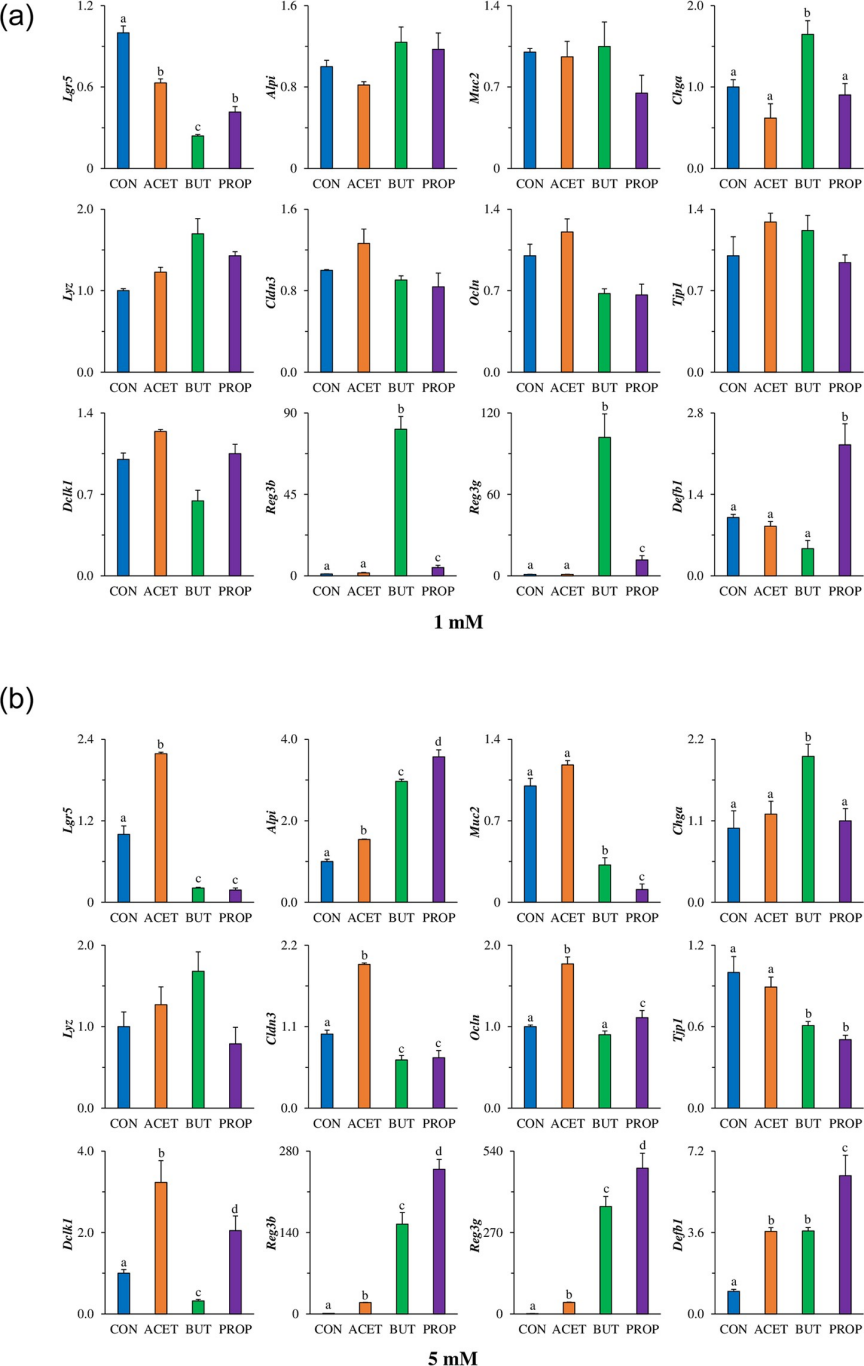
Mouse enteroids treated with single SCFAs alters gene expression after 24h. Effect of 24 h treatment with acetate (ACET), butyrate (BUT), or propionate (PROP) at (A) 1 mM and (B) 5 mM. Gene expression of differentiation markers: stem cells (*Lgr5)*, enterocyte alkaline phosphatase (*Alpi*), goblet cell mucin 2 (*Muc2*), enteroendocrine cell chromogranin A (*Chga*), Paneth cell lysozyme (*Lyz*), tight junction markers claudin-3 (*Cldn3*), occludin (*Ocln*), zonula-occludens 1 (*Tjp1*), Tuft cell marker *Dclk1*, anti-microbial peptide expression *Reg3β*, *Reg3γ*, and beta-defensin 1 (*Defb1*) were analyzed in murine 3D duodenal enteroids. Treatments are compared to controls (CON) which are set to 1.0 and superscripts (a,b,c,d) indicate statistical significance between treatments at *P* < 0.05, n = 4.

Duodenal murine enteroids were exposed to 1 and 5 mM SCFAs for 24 h. Expression of stem cell marker leucine-rich repeat-containing G-protein coupled receptor 5 (*Lgr5*) was increased more than 2-fold in murine enteroids treated with 5 mM ACET (*P* < 0.05). Conversely, enteroids treated with 5 mM BUT, or PROP had greater than 5-fold decreases in *Lgr5* compared to CON (*P* < 0.05; Fig 2b). There was no difference in *Lgr5* expression in murine enteroids treated with 1 mM SCFAs (*P* > 0.05; Fig 2a). Enterocyte biomarker alkaline phosphatase (*Alpi*) was significantly increased in response to all SCFA treatments but was highest (3.5-fold; *P* < 0.05) in enteroids treated with PROP. There was no difference in *Alpi* expression in murine enteroids treated with 1 mM SCFAs (*P* > 0.05; Fig 2a). Gene expression of goblet cell marker mucin 2 (*Muc2*) was not significantly affected by ACET treatment, however, *Muc2* was significantly decreased due to both BUT and PROP treatment (3-fold and 10-fold respectively; *P* < 0.05). *Muc2* expression was not altered in murine enteroids treated with 1 mM SCFAs (*P* > 0.05; Fig 2a). Paneth cell marker lysozyme (*Lyz*) had a tendency (*P* = 0.08) to increase due to BUT treatment but there was no effect observed when treated with 1 mM SCFAs (*P* > 0.05; Fig 2a). Enteroendocrine cell marker chromogranin A (*Chga*) was only significantly increased in BUT treated enteroids at both 1 mM and 5 mM concentrations (*P* < 0.05). Tuft cell marker doublecortin-like kinase 1 (*Dclk1*) was significantly elevated in response to 5 mM ACET (3-fold) and PROP (2-fold) treatments compared to CON (*P* < 0.05; Fig 2b). There was no difference in *Dclk1* expression in murine enteroids treated with 1 mM SCFAs (*P* > 0.05; Fig 2a). Antimicrobial peptide gene expression of c-type lectins regenerating islet-derived protein 3β (*Reg3β*) and 3γ (*Reg3γ*; Fig 2b) were increased moderately due to ACET treatment (20-fold and 40-fold), but were much more significantly increased due to BUT (150-fold and 350-fold, respectively) and PROP (250-fold, and 480-fold, respectively, *P* < 0.05) compared to CON. Only 1 mM BUT increased Reg3β and Reg3γ expression (80-fold and 100-fold respectively; *P* < 0.05). Antimicrobial peptide, β-defensin 1 (*Defb1*), was increased 3.5-fold due to ACET and BUT treatment, and 6-fold due to PROP treatment (*P* < 0.05); Fig 2b). 1 mM PROP was sufficient to increase *Defb1* expression 2-fold compared to CON (*P* < 0.05). Tight junction markers occludin (*Ocln*), and claudin-3 (*Cldn3*) were increased nearly 2-fold in enteroids treated with 5 mM ACET (*P* < 0.05). BUT decreased *Cldn3* expression at 5 mM but *Ocln* was not altered (*P* > 0.05). 5 mM PROP significantly decreased *Cldn3* (*P* < 0.05). There was no difference in *Cldn3* or *Ocln* expression in murine enteroids treated with 1 mM SCFAs (*P* > 0.05; Fig 2a). Tight junction protein 1 (*Tjp1*) was not altered by ACET treatment, but was decreased 1.7 and 2-fold respectively due to BUT and PROP treatment (*P* < 0.05; Fig 2b). There was no difference in *Tjp1* expression in murine enteroids treated with 1 mM SCFAs (*P* > 0.05; Fig 2a).

### 3D human enteroid proliferation is affected by SCFA treatment

**Fig 3:**
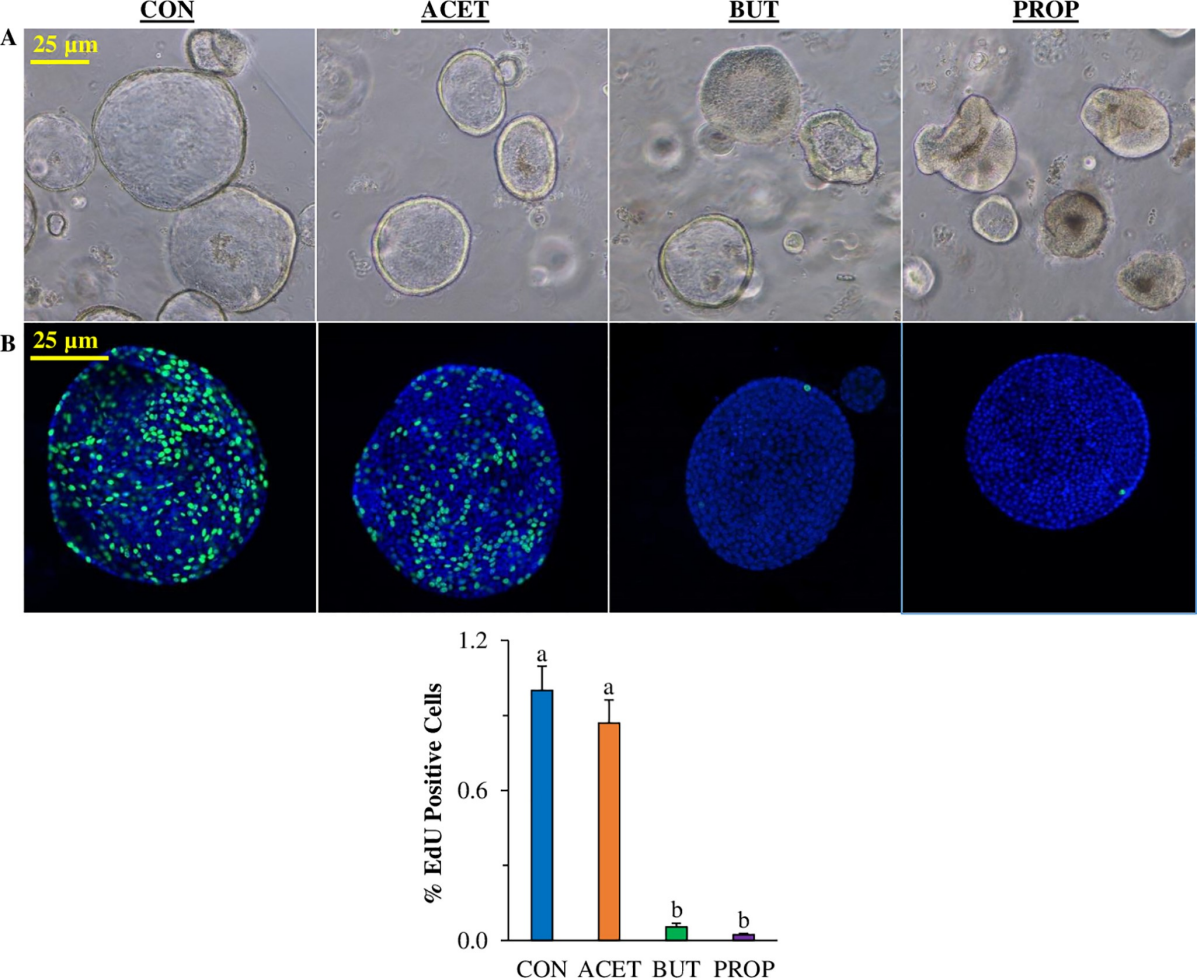
Effect of 24 h treatment with 10 mM acetate (ACET), butyrate (BUT), or propionate (PROP) on morphology of 3D human duodenal enteroids (A) and 3D human small intestinal enteroid cell proliferation (EdU staining, green) compared to controls (CON). Nuclei are stained with DAPI (blue) and images are representative of several fields of view at 20X magnification (B). Treatments are compared to controls (CON) which are set to 1.0 and superscripts (a,b,c,d) indicate statistical significance between treatments at *P* < 0.05, n = 6.

Human duodenal enteroids treated with 10 mM SCFAs exhibited altered cell proliferation as measured by EdU incorporated. The relative number of proliferating cells as determined by EdU staining which stains cells in the S phase of the cell cycle was significantly reduced in 3D human enteroids treated with 10 mM BUT or PROP (Fig 3b; *P* < 0.05). EdU was nearly undetectable in both treatments. However, ACET had no significant effect on cell proliferation compared to CON (*P* > 0.05).

### Gene expression in 3D human duodenal enteroids

**Fig 4:**
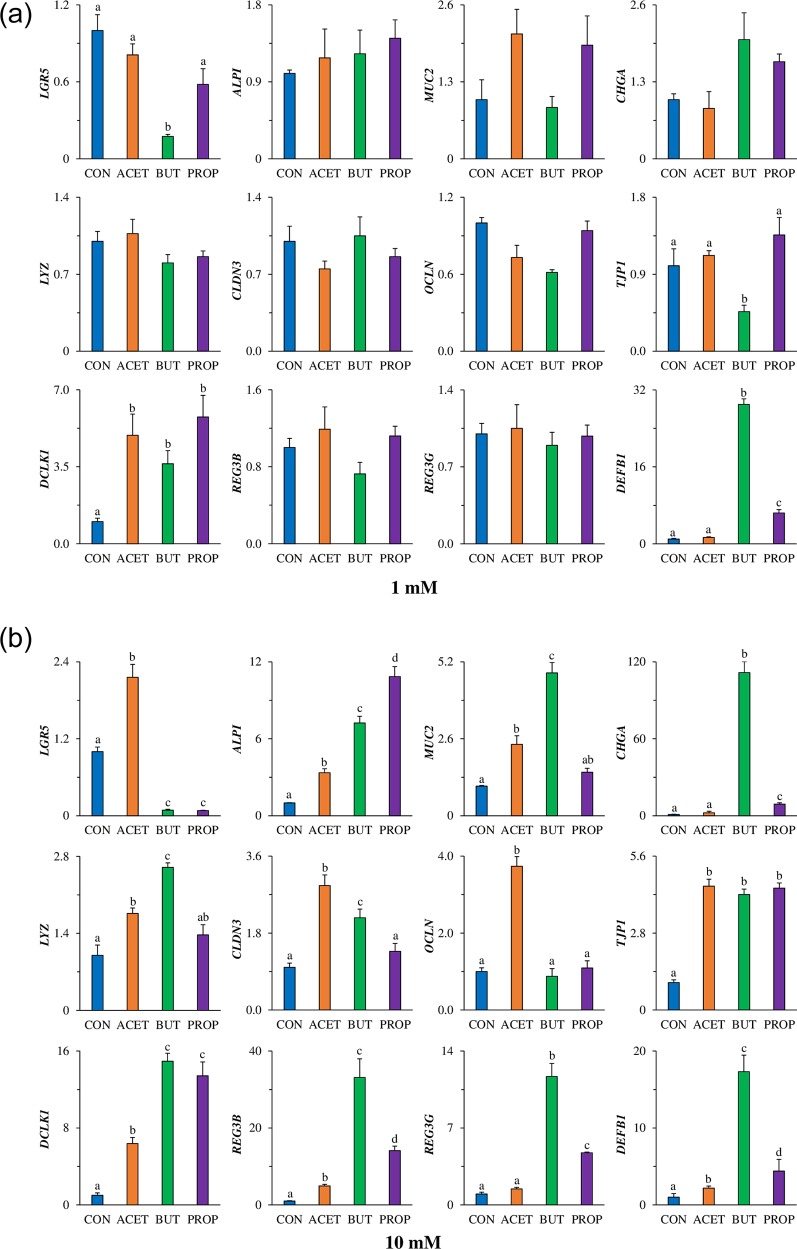
Effect of 24 h treatment with acetate (ACET), butyrate (BUT), or propionate (PROP) at (A) 1 mM and (B) 10 mM. Gene expression of differentiation markers: stem cells (*Lgr5)*, enterocyte alkaline phosphatase (*Alpi*), goblet cell mucin 2 (*Muc2*), enteroendocrine cell chromogranin A (*Chga*), Paneth cell lysozyme (*Lyz*), tight junction markers claudin-3 (*Cldn3*), occludin (*Ocln*), zonula-occludens 1 (*Tjp1*), Tuft cell marker *Dclk1*, anti-microbial peptide expression *Reg3β*, *Reg3γ*, and beta-defensin 1 (*Defb1*) were analyzed in human 3D duodenal enteroids. Treatments are compared to controls (CON) which are set to 1.0 and superscripts (a,b,c,d) indicate statistical significance between treatments at *P* < 0.05, n = 4.

Gene expression of *LGR5* was increased more than 2-fold in human duodenal enteroids treated with 10 mM ACET. Conversely, enteroids treated with 10 mM BUT and PROP had a greater than 10-fold decrease in *LGR5* compared to controls (CON; *P* < 0.05; Fig 4b). Only 1 mM treatment of BUT was able to significantly decrease *LGR5* expression (*P* < 0.05; Fig 4a).

Treatment with 10 mM SCFA significantly increased *ALPI* expression treatments but was highest (11-fold) in enteroids treated with PROP. There was no difference in *ALPI* expression in enteroids treated with 1 mM SCFAs (*P* > 0.05). Gene expression of *MUC2* was increased 2-fold due to ACET treatment and 5-fold due to BUT treatment (*P* < 0.05; Fig 4b) but was not increased due to PROP treatment. There was no difference in *MUC2* expression in enteroids treated with 1 mM SCFAs (*P* > 0.05). Lysozyme (*LYZ*) increased due to BUT alone (*P* < 0.05) however there was no difference in *ALPI* expression in enteroids treated with 1 mM SCFAs (*P* > 0.05). Enteroendocrine cell marker *CHGA* was significantly increased (170-fold) in BUT treated enteroids. There was no difference in *CHGA* expression in enteroids treated with 1 mM SCFAs (*P* > 0.05). Tuft cell marker *DCLK1* was significantly elevated due to ACET (7-fold) and BUT (14-fold) treatment compared to CON (*P* < 0.05; Fig 4b). 1 mM ACET, BUT and PROP significantly increased *DCLK1* expression (5-fold, 3.6-fold, and 5.8-fold, respectively; *P* < 0.05). Anti-microbial peptide gene expression of *REG3β* and *REG3γ* (Fig 4b) were not increased due to ACET treatment, however, were significantly increased due to BUT (2.5-fold and 3-fold, respectively) compared to CON. There was no difference in *REG3β* and *REG3γ* expression in enteroids treated with 1 mM SCFAs (*P* > 0.05). β-defensin 1 (*DEFB1*) was increased 75-fold and 66-fold respectively in BUT and PROP treated enteroids (*P* < 0.05; Fig 4b). A similar pattern was observed for 1 mM BUT and PROP (*P* < 0.05). Tight junction marker expression of *OCLN* was only significantly elevated in ACET treated enteroids (4-fold; *P* < 0.05, Fig 4b). There was no difference in *OCLN* expression in enteroids treated with 1 mM SCFAs (*P* > 0.05). However, *CLDN3* and *TJP1* were increased due to both ACET and BUT treatments (*P* < 0.05, Fig 4b). There was no difference in *CLDN3* expression in enteroids treated with 1 mM SCFAs (*P* > 0.05), however 1 mM BUT decreased TJP1 expression more than 2-fold (*P* < 0.05).

### 2D human duodenal enteroid proliferation is affected by SCFA treatment

**Fig 5:**
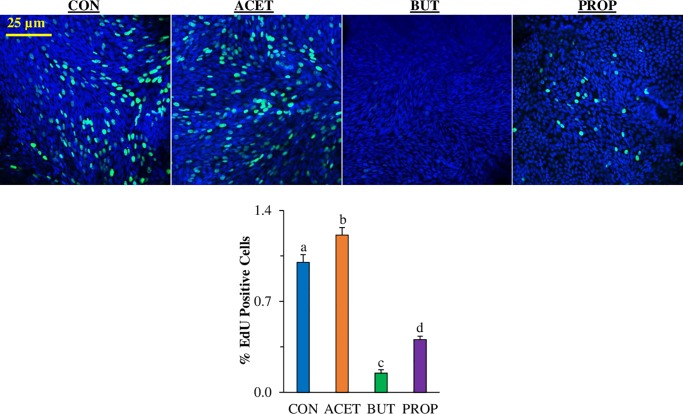
Effect of 24 h treatment with 10 mM acetate (ACET), butyrate (BUT), or propionate (PROP) on 3D (a) and 2D (b) human small intestinal enteroid cell proliferation (EdU staining, green) compared to controls (CON). Nuclei are stained with DAPI (blue) and images are representative of several fields of view at 20X magnification. Treatments are compared to controls (CON) which are set to 1.0 and superscripts (a,b,c,d) indicate statistical significance between treatments at *P* < 0.05, n = 6.

2D human enteroids treated with 10 mM BUT and PROP had a significant decrease in proliferation as measured by the EdU proliferation assay (7-fold and 2.5-fold respectively; Fig 5). Conversely, 2D enteroids treated with 10 mM ACET had increased proliferation (*P* < 0.05).

### Gene expression and protein secretion in 2D human duodenal enteroids

**Fig 6:**
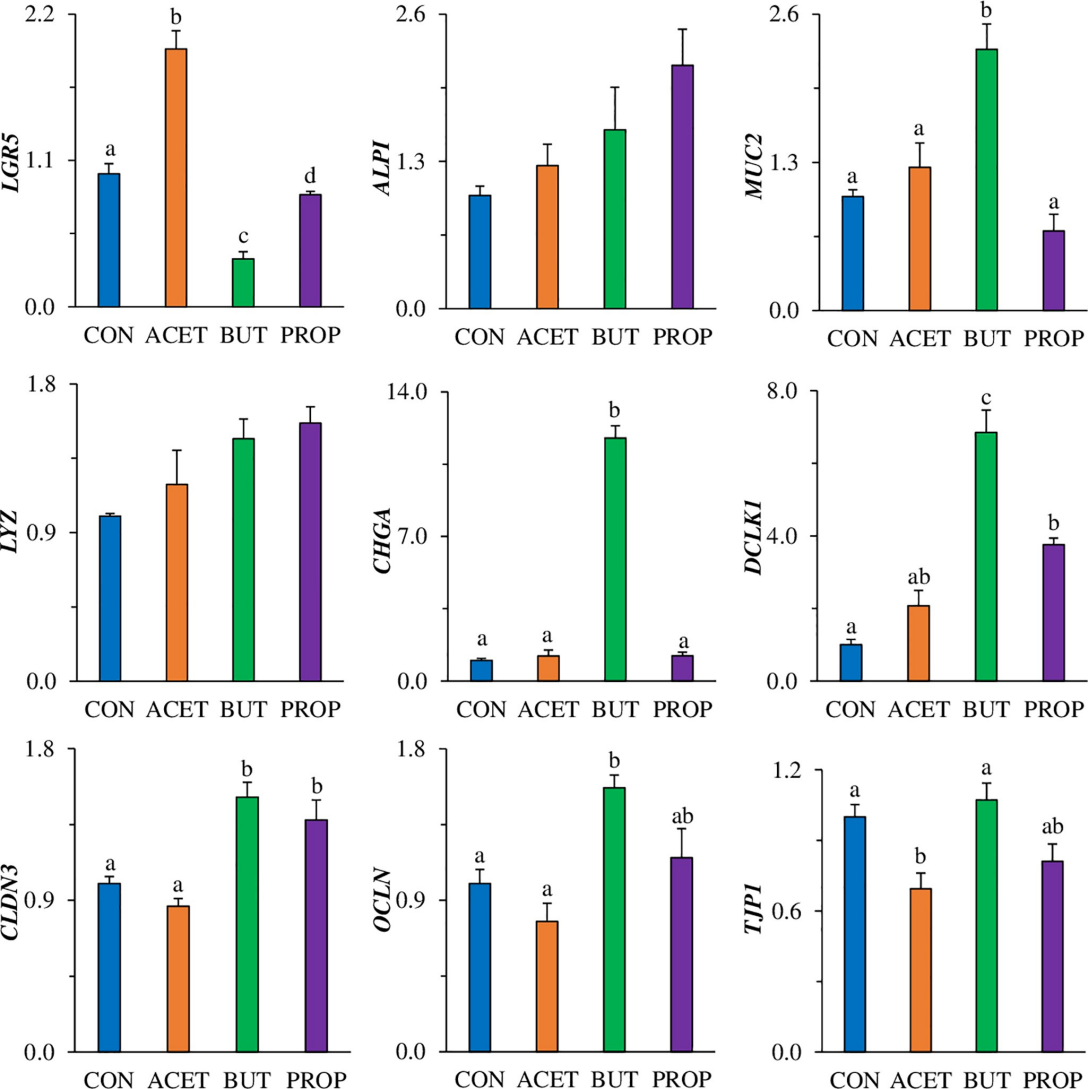
Effect of 24 h treatment with acetate (ACET), butyrate (BUT), or propionate (PROP) at 10 mM. Gene expression of differentiation markers: stem cells (*Lgr5)*, enterocyte alkaline phosphatase (*Alpi*), goblet cell mucin 2 (*Muc2*), enteroendocrine cell chromogranin A (*Chga*), Paneth cell lysozyme (*Lyz*), tight junction markers claudin-3 (*Cldn3*), occludin (*Ocln*), zonula-occludens 1 (*Tjp1*), Tuft cell marker *Dclk1*, anti-microbial peptide expression *Reg3β*, *Reg3γ*, and beta-defensin 1 (*Defb1*) were analyzed in human 2D duodenal enteroids. Treatments are compared to controls (CON) which are set to 1.0 and superscripts (a,b,c,d) indicate statistical significance between treatments at *P* < 0.05, n = 4.

*LGR5* expression was significantly increased (2-fold; *P* < 0.05) in enteroids treated with 10 mM ACET (Fig 6) while enteroids treated with 10 mM BUT had significantly decreased expression of *LGR5* (2.8-fold; *P* < 0.05). PROP slightly decreased *LGR5* expression. *ALPI* expression in 2D enteroid monolayers was not affected by treatment (*P* > 0.05), however there was a similar trend as in the human 3D enteroids. *MUC2* expression was significantly increased (2.3-fold; *P* < 0.05) by 10 mM BUT treatment alone. *LYZ* expression was not significantly altered by 10 mM SCFAs in 2D enteroid monolayer (*P* > 0.05). *CHGA* expression was specifically, and significantly increased by 10 mM BUT (11.8 fold; *P* < 0.05). Pattern was similar to that observed in both 3D murine, and 3D human enteroids, however, magnitude was significantly less. *DCLK1* was significantly increased by both 10 mM BUT and 10 mM PROP (6.8-fold and 3.8-fold, respectively; *P* < 0.05) compared to CON (Fig 6). *REG3β*, *REG3γ*, and *DEFB1* gene expression were not detectable in 2D human enteroid monolayers. Tight junction related marker claudin-3 (*CLDN3*) expression was significantly increased by 10 mM BUT and 10 mM PROP (1.5 and 1.4-fold, respectively; *P* < 0.05). Occludin (*OCLN*) expression was significantly increased by 10 mM BUT treatment alone (*P* < 0.05). *TJP1* expression was not affected by 10 mM BUT or PROP expression, however was actually decreased by 10 mM ACET (1.5 fold; *P* < 0.05).

### Protein expression and secretion in 2D human duodenal enteroids

Cell supernatants as well as media from the basolateral side of Transwell^™^ inserts were analyzed for enteroendocrine hormones as well as cytokines. As was expected, cell supernatants overall had higher expression of all proteins analyzed (Table 1). Enteroendocrine hormone peptide YY (PYY) was not significantly increased in cell supernatant, however, there was a tendency for it to increase in 10 mM BUT treated enteroids (*P* = 0.08). It was however, significantly increased in the basolateral media (*P* < 0.05; Table 1). Ghrelin was significantly increased by all SCFAs in cell supernatants, with BUT and PROP showing highest concentrations (*P* < 0.05). Ghrelin was also significantly increased by 10 mM BUT alone in basolateral media (*P* < 0.05; Table 1).

**Table 1:** Protein expression of neuroendocrine hormones and cytokines in human 2D duodenal enteroids treated with short chain fatty acids.

Cell Supernatant	CON	ACET	BUT	PROP	*P*-value
Peptide YY, pg/mg	760 ± 6.3	780 ± 74.7	956 ± 32.0	778 ± 17.6	0.08
Ghrelin, ng/mg	82^a^ ± 0.9	96^b^ ± 2.3	109^c^ ± 3.6	105^bc^ ± 2.4	<0.05
Interleukin 4, pg/mg	833^a^ ± 10.9	893^a^ ± 80.0	1151^b^ ± 26.8	1117^b^ ± 49.3	<0.05
Interleukin 8, pg/mg	232^ab^ ± 6.8	234^ab^ ± 3.5	306^a^ ± 8.0	260^b^ ± 2.7	<0.05
Interleukin 18, pg/mg	827 ± 4.8	837 ± 33.1	1029 ± 66.6	982 ± 10.6	0.08
sRANKL, pg/mg	455^a^ ± 4.0	442^a^ ± 6.4	638^b^ ± 34.8	503^a^ ± 18.4	<0.05
**Basolateral Media**					
Peptide YY, pg/mg	223^a^ ± 10.9	193^a^ ± 23.8	287^b^ ± 5.0	212^a^ ± 0.4	<0.05
Ghrelin, ng/mg	22^a^ ± 1.7	19^a^ ± 1.1	31^b^ ± 1.8	20^a^ ± 1.0	<0.05
Interleukin 4, pg/mg	243^a^ ± 17.9	195^a^ ± 0.9	368^b^ ± 28.	226^a^ ± 10.4	<0.05
Interleukin 8, pg/mg	1119^a^ ± 43.7	1155^a^ ± 15.0	1280^b^ ± 8.8	1058^c^ ± 53.5	<0.05
Interleukin 18, pg/mg	134^a^ ± 13.8	125^a^ ± 6.0	218^b^ ± 19.2	132^a^ ± 4.7	<0.05
sRANKL, pg/mg	111^a^ ± 11.2	82^a^ ± 2.5	196^b^ ± 18.3	111 ^a^ ± 4.0	<0.05

Cells were normalized to protein concentration. 2D human enteroid monolayers were treated with 10 mM acetate (ACET), butyrate (BUT), and propionate (PROP) for 24 h. Treatments are compared to controls (CON) and superscripts (a,b,c,d) indicate statistical significance between treatments at *P* < 0.05, n = 3.

**Fig 7:**
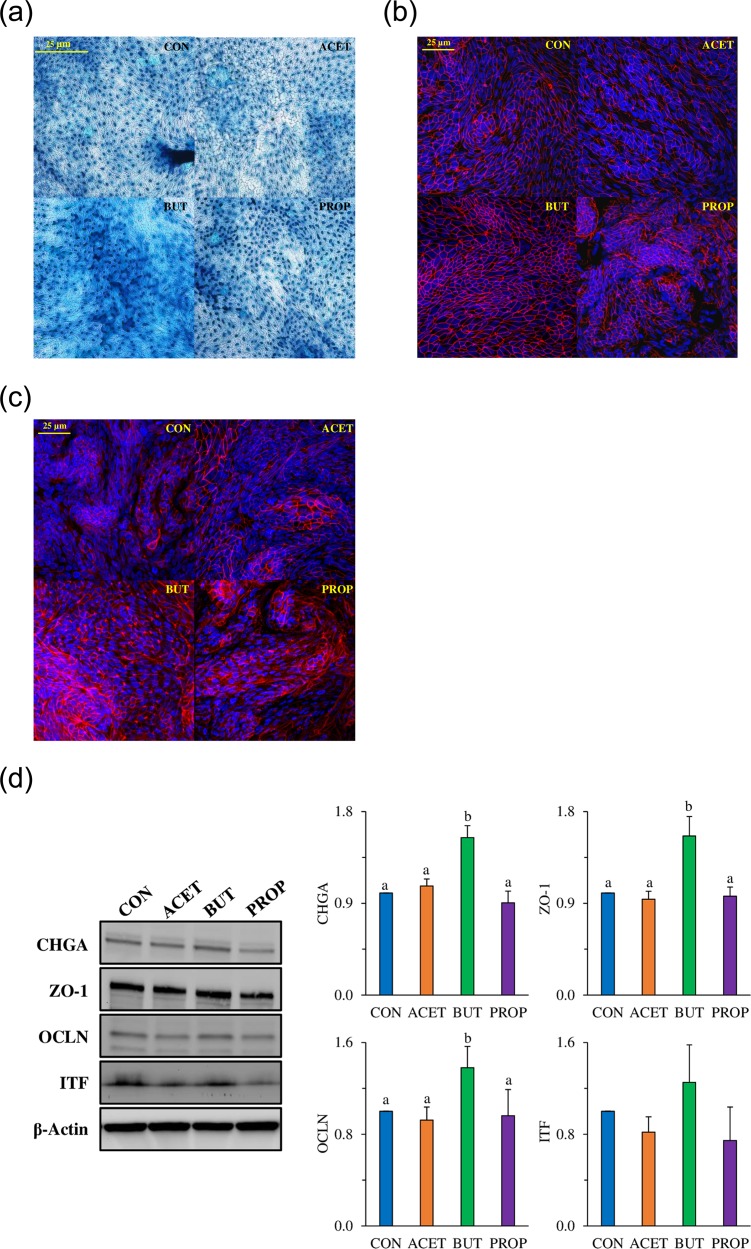
(A) Alcian blue/Hematoxylin staining 2D enteroids treated with 10 mM acetate (ACET), butyrate (BUT), and propionate (PROP) compared to controls (CON). (B) Zonula occludens-1 (ZO-1) staining (red) and (C) Claudin-3 (CLDN3) staining (red). Nuclei are stained with DAPI (blue). (D) Western blot data for Chromogranin-A (CHGA), Zonula-occludens-1 (ZO-1), occludin (OCLN), and Trefoil Factor 3 (TFF3). Samples were compared to β-actin as a housekeeping protein. Confocal images are representative of several fields of view at 40X magnification. Treatments are compared to controls (CON) which are set to 1.0 and superscripts (a,b,c,d) indicate statistical significance between treatments at *P* < 0.05, n = 4.

Interleukin 4 was significantly increased in 10 mM BUT and PROP treated 2D enteroids (P < 0.05) and was only increased significantly in basolateral media by 10 mM BUT (*P* < 0.05; Table 1). Interleukin 8 was significantly increased in cell supernatants of 10 mM BUT treated enteroids as well as basolateral media (*P* < 0.05). Interleukin 18 was not significantly different in cell supernatant, however, there was a tendency for 10 mM BUT to increase expression (*P* = 0.08; Table 1). Interleukin 18, however was increased by 10 mM BUT treatment in basolateral media (*P* < 0.05). Soluble RANK Ligand (sRANKL) was significantly increased in 10 mM BUT treated enteroid cell supernatant and basolateral media (*P* < 0.05; Table 1). 2D human duodenal enteroid monolayers treated with 10 mM SCFAs were analyzed for mucin expression utilizing a histological stain, Alcian blue/hematoxylin (Fig 7a). Monolayers express and secrete mucin but this appears to be higher in BUT treated samples, however this was not quantifiable. In addition, SCFA-treated monolayers were stained with specific tight junction markers, zonula occludens-1 (ZO-1; Fig 7b) and claudin-3 (CLDN3; Fig 7c) which showed objectively higher amounts in BUT treated enteroids. Western blotting of ZO-1 and CHGA produced a 1.55-fold increase protein expression in BUT treated enteroids compared to CON (*P* < 0.05) while ACET and PROP were not different. OCLN expression was also increased 1.38-fold in BUT treated enteroids compared to CON. OCLN blot had multiple bands, indicating either post-translation modification or varying isoforms. Most prominent banding was utilized for protein quantification (S1 Raw image). However, ITF was not statistically different across treatments. (*P* > 0.05).

### Butyrate and propionate increase transepithelial electrical resistance in human 2D duodenal enteroids

**Fig 8:**
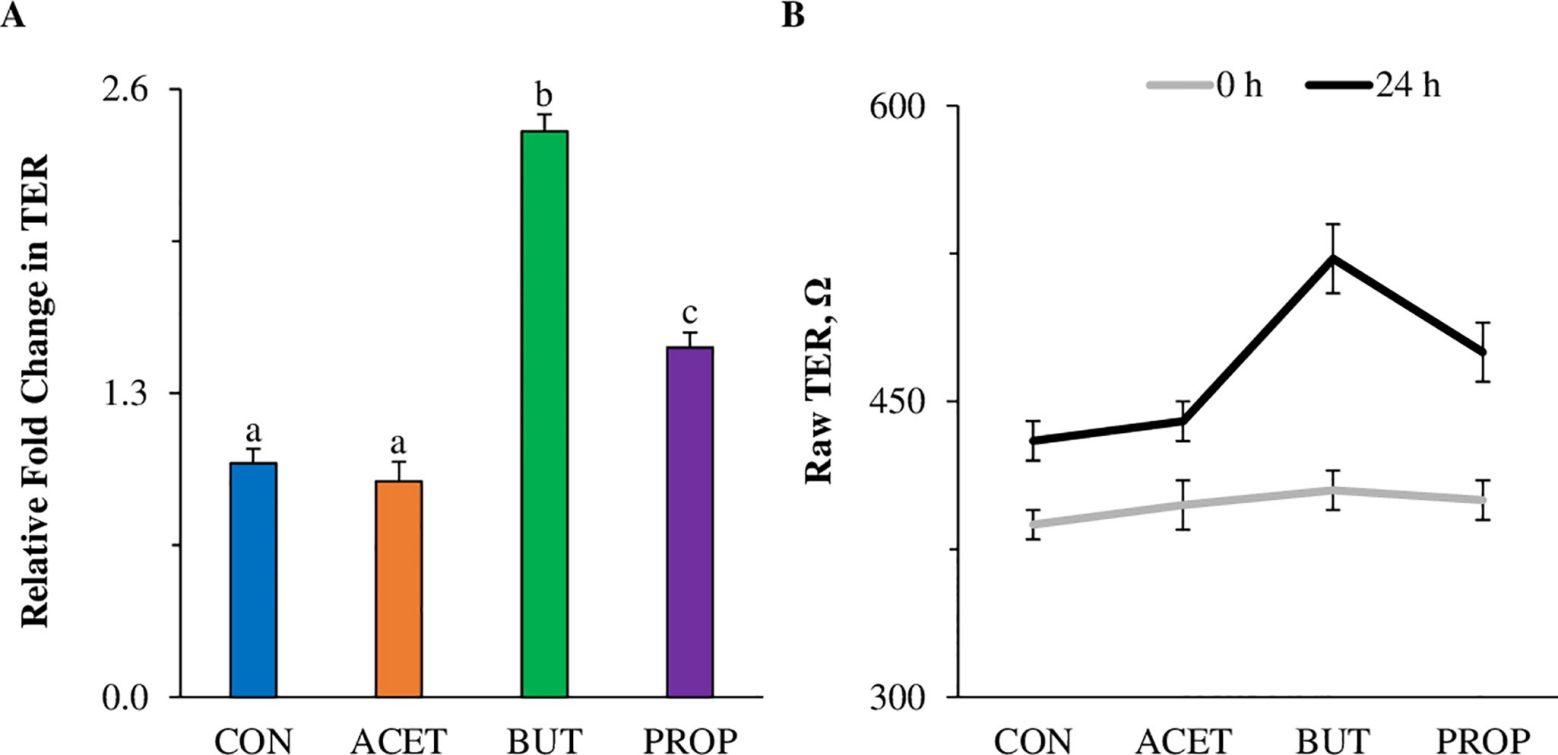
Effect of 24 h treatment with 10 mM acetate (ACET), butyrate (BUT), or propionate (PROP) on (A) epithelial barrier function of human duodenal enteroids as measured by TER compared to controls (CON). Values are represented as relative change in TER at time 0 and time 24 h after treatment. (B) Raw TER values at 0 h and 24 h post-treatment. Treatments are compared to controls (CON) which are set to 1.0 and superscripts (a,b,c,d) indicate statistical significance between treatments at *P* < 0.05, n = 4.

2D enteroids treated with 10 mM BUT had a 2.4-fold increase in relative Transepithelial electrical resistance (TER) compared to CON enteroids (Fig 8a; *P* < 0.05) after 24 h of treatment. PROP also increased TER (1.5-fold; *P* < 0.05), while ACET treated enteroids were not different from CON (Fig 8a; *P* > 0.05). All enteroids, regardless of treatment. exhibited increased TER from 0–24 h (Fig 8a).

## Discussion

Usage of the enteroid model to study host-microbe and host-microbial metabolite interactions is growing [18, 22]. Previous *in vitro* and *in vivo* research has shown the beneficial properties of SCFAs on the intestinal epithelium [23]. However, previous *in vitro* models lacked the cellular complexity of enteroids which contain all epithelial cell types at relative proportions found *in vivo*. Specifically, how SCFAs modulate the distribution of intestinal epithelial cell types and modulate intestinal stem cell lineages is not fully understood. In addition, BUT has been the most widely studied SCFA, while less information is available on ACET and PROP, which represent a larger fraction of the total SCFA pool in the intestine. Few studies to date have examined microbial metabolites in the enteroid model, however, this area has shown increased attention in recent years. For these experiments, we took a progressive approach by first examining SCFA effects in a 3D murine model, then taking information gleaned from that to move into a human 3D model. Finally, we wanted to examine functional and protein changes using a 2D human monolayer model.

In the current study, gene expression stem cell marker *Lgr5* and cellular proliferation marker EdU were dramatically decreased due to exposure to BUT and PROP in both mouse and human enteroids. This was highly reproducible and translatable between 2D and 3D models and was similar to previous findings in intestinal *in vitro* models [9, 24]. In our model, ACET increased proliferation in both mouse and human enteroids only at 10 mM. Previous research examining cell proliferation in gastric adenocarcinoma epithelial cells showed that ACET (up to 12.5 mM) increased cell viability and proliferation while >12.5 mM inhibited cell growth [25]. Additionally, another study showed that ACET induced cell proliferation arrest at 20 mM in murine small intestinal crypt cell line m-ICcl2 [26]. Effects of SCFAs on proliferation appear to be largely dependent on concentration as we also tested 1 mM SCFAs in murine enteroids and found no effect on cellular proliferation. It is interesting to note that normally stem cells are not exposed to elevated concentrations of SCFAs *in vivo* as most of the SCFAs produced (especially BUT) are taken up and utilized by villus-residing cells [9].

Enterocyte differentiation marker *Alpi* was increased with exposure to all SCFAs, especially BUT and PROP. This is consistent with previous results that have shown potent anti-proliferative HDAC activity of BUT and PROP [27, 28]. SCFAs have also been shown to stimulate mucin production and secretion [29, 30]. Goblet cell marker, *Muc2* gene expression was increased due to ACET and BUT in human 3D enteroids, but was conversely decreased in mouse 3D enteroids. Previous studies have shown BUT to cause increased mucus secretion which would be consistent with our current human data [31, 32]. Alcian blue/hematoxylin staining was also conducted on 2D human enteroids. Alcian blue is a histological stain for mucins found in intestinal goblet cells. Results showed moderate visual differences in staining intensity in BUT treated enteroids.

Trefoil factor 3 (TFF3) is a protein that is known to be expressed in intestinal goblet cells. TFFs are known to play a role in epithelial restitution and TFF3 has been correlated with down-regulation of inflammatory cytokines [33]. Although TFFs have been highly associated with goblet cells and mucus secretion, little is known about how short-chain fatty acids affect its expression. Tran et al. observed that expression of TFF3 was potently and dose-dependently inhibited by short-chain fatty acids, with BUT having the highest inhibitory effect [34]. In the current study, TFF3 gene and protein expression (both cell supernatant and basolateral media) in 2D enteroids were significantly decreased by BUT and PROP (S2 Fig), thus eludes to an effect of differentiation. Interestingly, 3D TFF3 gene expression was increased by BUT in 3D and not changed in 3D media, which may be due to the polarity difference in the two models.

Most interestingly, mRNA expression of enteroendocrine cell marker, chromogranin A (*Chga)* was increased in both mouse and human enteroids exposed specifically to BUT, in both 2 and 3D formats. Chromogranin A is a non-specific neuroendocrine cell marker which is a major component of secretory granules of several endocrine cell types found in the intestine. Enteroendocrine cells (EEC) comprise less than 1% of the total epithelial cell population, however have various important functions including chemosensing, satiety hormone production, and secretion, including glucagon-like peptide-1 (GLP-1), peptide YY (PYY), cholecystokinin (CCK), and gastrin, among others.

In the current study, secretion of PYY protein into the cell media was stimulated by all SCFA treatments, with BUT having the greatest effect. Previous work has shown that 5 mM BUT stimulates PYY production in enteroendocrine cell lines. High SCFA concentrations have been associated with elevated plasma levels of PYY and GLP-1 via increased hormone secretion and/or increased number of EEC’s [35, 36]. Larraufe et al. showed increased expression of the PYY gene in enteroendocrine cell lines when treated with 2 mM ACET, PROP, or BUT, with BUT being the largest inducer of expression. The same researchers also incubated human colonic primary cultures with SCFAs and also observed an increase in the expression of PYY: BUT and PROP increased expression ~16-fold and ~7-fold respectively, whereas ACET increased expression ~2-fold. Interestingly, Tsuruta et al. showed a moderate increase in *CHGA* mRNA with 0.1 and 1 mM ACET but not 0.1 and 1mM PROP or 0.1 mM BUT in colonic enteroids, and this was correlated with increased *CHGA* protein expression as measured by immunofluorescence and may be a colon-specific response [20]. We were able to verify CHGA protein results by Western blot.

Chromogranin A is also a marker of ghrelin-secreting cells. Ghrelin which is a hunger-inducing hormone, is largely found in the stomach, however it also has been shown to be secreted from enteroendocrine cells in the duodenum [37]. Ghrelin has previously been detected in human intestinal enteroid models [38]. Interestingly, a previous study showed that BUT stimulated ghrelin expression in human gastric-cancer cells [39]. In the current study, we observed a significant increase in ghrelin in the protein supernatant due to all SCFAs, but in the basolateral media the response was specific to BUT alone. Taken together, these data suggest that enteroids express functional enteroendocrine cells that can sense and respond to signals.

Tuft cell marker *Dclk1* was also increased most significantly by BUT and PROP, but also by ACET in human enteroids while ACET induced the highest change in expression in murine enteroids. Tuft cells are a relatively new epithelial sub-type that represent approximately 0.4 to 2% of the total cell population [40]. They are thought to promote cell proliferation, may act as reserve stem cells, may enhance epithelial barrier integrity [41] and perhaps are most well-known for their ability to activate the immune system, specific to type 2 innate immunity [42]. In the current study, an increase in expression of *Dclk1* may be a response to decreased stem cell proliferation caused by SCFA exposure.

Paneth cell marker and anti-microbial marker *Lyz* was increased in all SCFA-treated enteroids with BUT having the highest effect. Paneth cells, which reside in the intestinal crypts of the small intestine, next to stem cells, sense and respond to pathogens by making and secreting several types of anti-microbial peptides [43]. Effects of SCFAs on Paneth cell function have not been directly studied, however the anti-microbial peptides they produce have been examined under SCFA exposure [44]. Antimicrobial proteins are produced and secreted by intestinal epithelial and Paneth cells and serve as an additional line of defense against pathogens while maintaining homeostasis with commensal bacteria. In the current study, *Reg3β* and *Reg3γ* gene expression was drastically induced by exposure to 5 mM BUT or PROP in mouse enteroids. In human enteroids, *REG3β* and *REG3γ* were significantly elevated by 10 mM BUT exposure in 3D but not 2D forms (not detectable by qPCR in 2D). Defensins are also expressed in epithelial Paneth cells, and β-defensins are the most highly expressed and have shown activity against gram positive as well as gram negative bacteria [45,46]. There are few studies showing how SCFAs affect defensin expression or function, however one study found that BUT induced human beta defensin-2 (HBD-2) gene expression in colorectal cancer cell lines [47] while another showed that β-defensins were upregulated by BUT in a porcine model and *in vitro*. Another study utilizing 3D human patient-derived enteroids showed that stimulation with 5 mM BUT significantly upregulated mRNA expression of *DEFB1* [48].

Short chain fatty acids have also been shown to have anti-inflammatory activity in immune cells [5, 7], however, effects on intestinal host cells are not well understood. Interleukins (ILs) are secreted cytokines that regulate immune responses and can function in a pro- or anti-inflammatory manner. Interleukin-4 is thought to have several functions including stimulation of B and T lymphocytes, macrophage polarization, as well as goblet and Tuft cell hyperplasia [49, 50]. An increase in IL-4 protein was detected along with increases in gene expression of *DCLK1* and *MUC2* for all SCFA treatments with the exception of *MUC2* in the PROP treated enteroids. Treatment with 20 mM SCFAs have been shown to induce IL-8, IL-6, and IL-1β release in neutrophils while concentrations below 2 mM had no effect [51]. Interleukin-8, which is a neutrophil chemoattractant, is a pro-inflammatory cytokine produced by epithelial cells and immune cells. Previous *in vitro* research showed that SCFAs regulate secretion of IL-8 from epithelial cells only in the presence of LPS [52], but we show in the current study that it can be stimulated by BUT exposure. Another cytokine, interleukin-18 was increased in the BUT and PROP treatments, however, while IL-18 has been previously reported to promote barrier function and intestinal homeostasis, it can also exacerbate damage induced by colitis [53, 54]. The IL-18 receptor is thought to be constitutively expressed by intestinal epithelial cells [55] and is a cytokine in the IL-1 family. In the current study IL-18 protein expression was increased in both cell supernatants and media. A previous enteroid study showed a significant increase in mRNA expression of IL-18 in response to 5 mM BUT exposure [51] and it has been implicated as a driving force in intestinal inflammatory disorders [55]. We also examined receptor activator of NF-κβ ligand (RANKL), which is a member of the tumor necrosis superfamily and is largely known for its role in bone remodeling, but it also has implications in M cell development [56] and BUT has been shown to increase RANKL expression in osteoblastic cells [57] and we observed an increase due to BUT treatment in cell supernatants and basolateral media in 2D human enteroid monolayers.

In several animal and human models, SCFAs have been shown to regulate epithelial tight junctions and enhance barrier function. For example, BUT upregulates tight junction proteins claudin-3 and 4 and enhances barrier function [58]. In addition, it has been shown to repress permeability promoting claudin-2 expression [6]. Mixtures of SCFAs also improve barrier function in a protective and reparative manner under LPS/TNF-α stimulation [59]. In 3D enteroids, tight junction gene expression of *OCLN* and *CLDN3* increased due to ACET exposure while *TJP1* was upregulated due to all SCFA treatments. In the 2D enteroid monolayer, BUT treatment, as well as PROP treatment increased barrier integrity as a function of TER.

In the current study, both 2D and 3D models were utilized for comparison as the morphology and structure of enteroids in each format is notably different, thus different responses might be expected. 3-dimensional enteroids are cultured in a basement membrane matrix droplet and have reversed polarity while 2-dimensional enteroids are cultured on a lightly collagen coated surface and have normal polarity. Wang et al. utilized 24 mM ACET, 1 mM BUT, and 6 mM PROP in 3D mouse colon and 2D human rectal monolayers. These researchers also compared 3D vs. 2D formats for use in dietary compound screening. They found that the general response of enteroids treated with many dietary compounds were similar, however, notable differences may have been due to physical complexity level of 3D enteroids due to their growth in a multi-planar hydrogel. An important distinction between models, aside from complexity, is polarity. Under normal growth conditions in a basement membrane matrix enteroids will self-assemble with the basolateral side out, and apical side facing inward thus effectively making them “inside out”. Many of the measures were similar between 2D and 3D human duodenal enteroids treated with SCFAs, but the most noticeable difference was in magnitude of expression changes. We speculate this may be due to a number of factors including complexity of the 3D environment in Matrigel, as well as reversed polarity. This would mean that SCFAs must be able to enter the lumen in order to exert effects normally and adding basolaterally may be similar to adding directly to the crypt compartment where highly sensitive cell types reside [15].

## Conclusions and future directions

Murine and human enteroids provide a physiologically relevant tool to study the role of bacterial metabolites in host health and function. This model has shown replicability from previous research examining effects of SCFAs on the intestine but has also provided new information regarding how other SCFAs aside from BUT affect intestinal cell function, especially of small intestine where SCFA concentrations are lower, but still present. At physiologically relevant concentrations, SCFAs have vast effects on all epithelial cell types and differentially affect cell lineages, as well as their function including pathogen defense, immune response, and intestinal barrier function. Future research in this area could examine other types of bacterial metabolites as well as incorporate more complex systems, such as immune cell and bacterial cell co-cultures, building upon the foundation we have laid in this study, and will aid in the further characterization of the effects of SCFAs on intestinal physiology.

## Supporting information

S1 Fig Gene and protein expression of trefoil factor 3 (TFF3) in 3D human enteroids and 2D enteroids treated with 10 mM acetate (ACET), butyrate (BUT) or propionate (PROP) compared to controls (CON) which are set to 1.0. Superscripts (a,b,c,d) indicate statistical significance at *P* < 0.05, n = 4.
(TIF)

S2 Fig Confocal microscopy z-stack cross-section of human small intestinal enteroid monolayers treated with 10 mM acetate (ACET) and 10 mM butyrate compared to controls. EdU (green), actin (red), nuclei (blue).
(TIF)

S1 Raw images Raw western blot images. (PDF)

S1 Table (DOCX)
