# Supplementary figures and images for: Intestinal enteroids recapitulate the effects of short-chain fatty acids on the intestinal epithelium
Source: PLoS One. 2020 Apr 2;15(4):e0230231. doi: 10.1371/journal.pone.0230231 (PMC7117711; doi:10.1371/journal.pone.0230231)

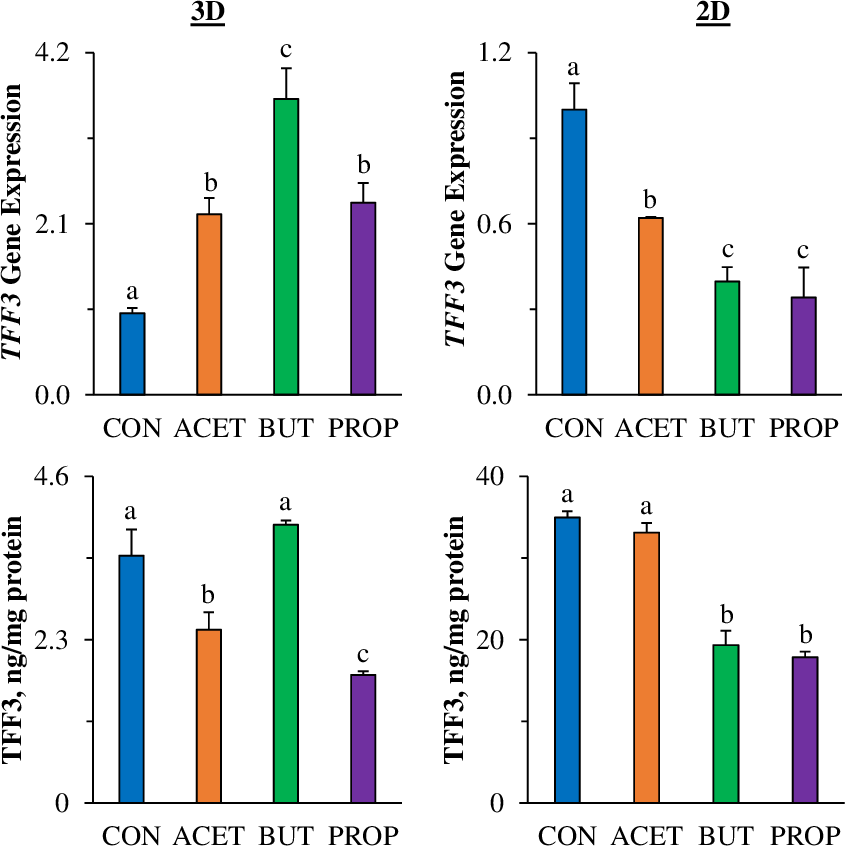

Supplement: S1 Fig — Superscripts (a,b,c,d) indicate statistical significance at P < 0.05, n = 4. (TIF) [file pone.0230231.s001.tif]

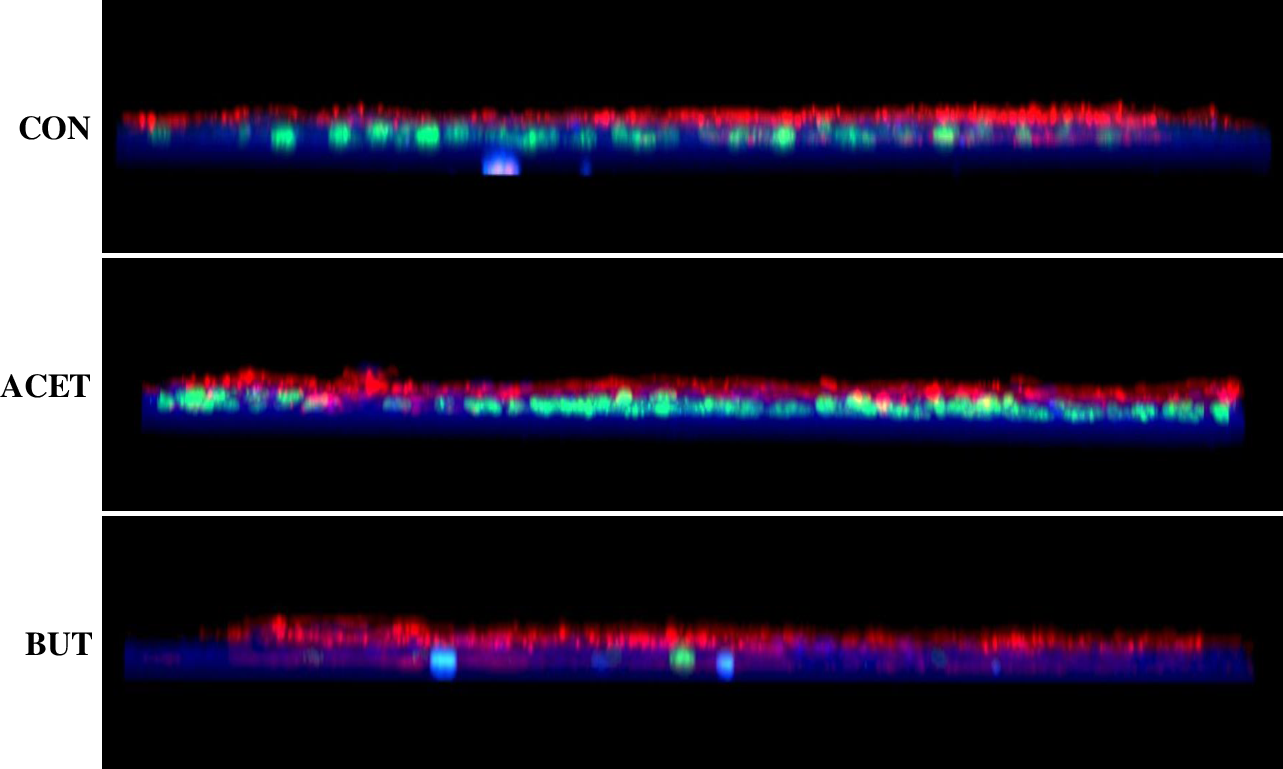

Supplement: S2 Fig — EdU (green), actin (red), nuclei (blue). (TIF) [file pone.0230231.s002.tif]
