# Supplementary material for: Intestinal enteroids recapitulate the effects of short-chain fatty acids on the intestinal epithelium
Source: PLoS One. 2020 Apr 2;15(4):e0230231. doi: 10.1371/journal.pone.0230231 (PMC7117711; doi:10.1371/journal.pone.0230231)
Supplement: S1 Raw images — (PDF) [file pone.0230231.s003.pdf]

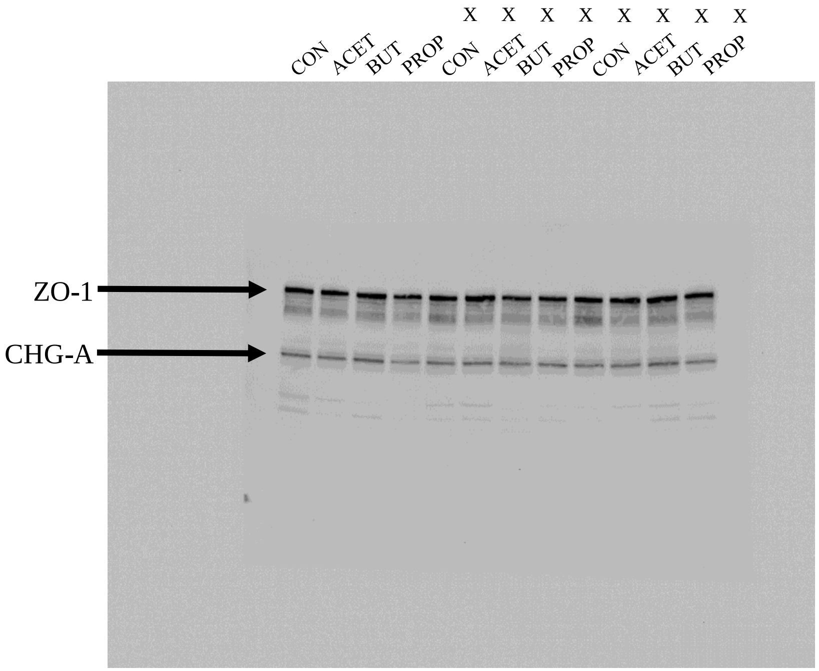

X – not used in final figure

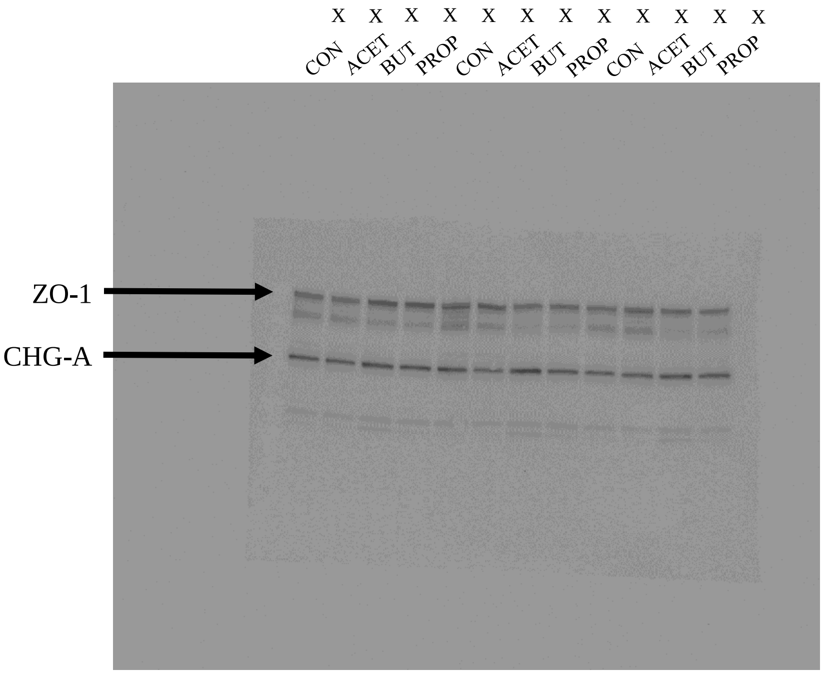

X – not used in final figure

Figure 7D

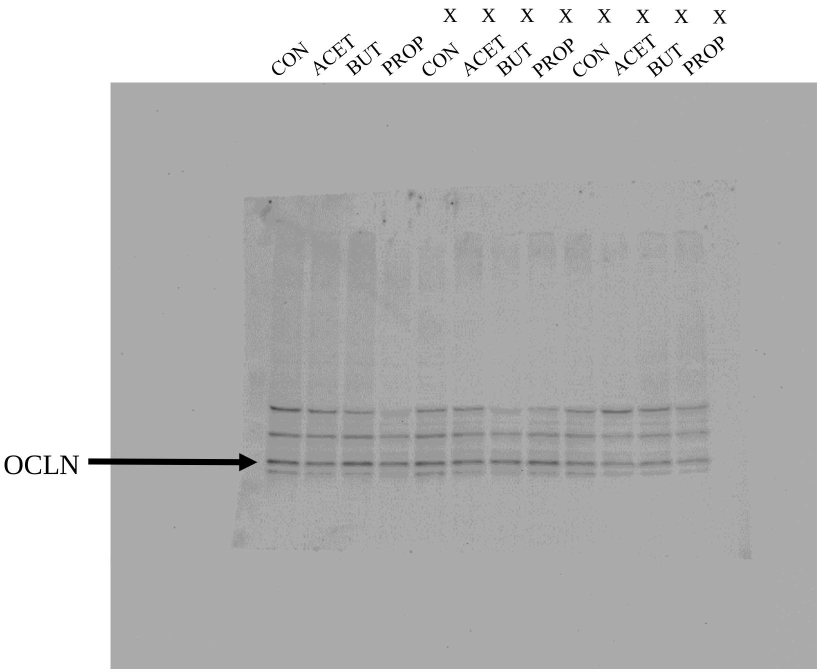

X – not used in final figure

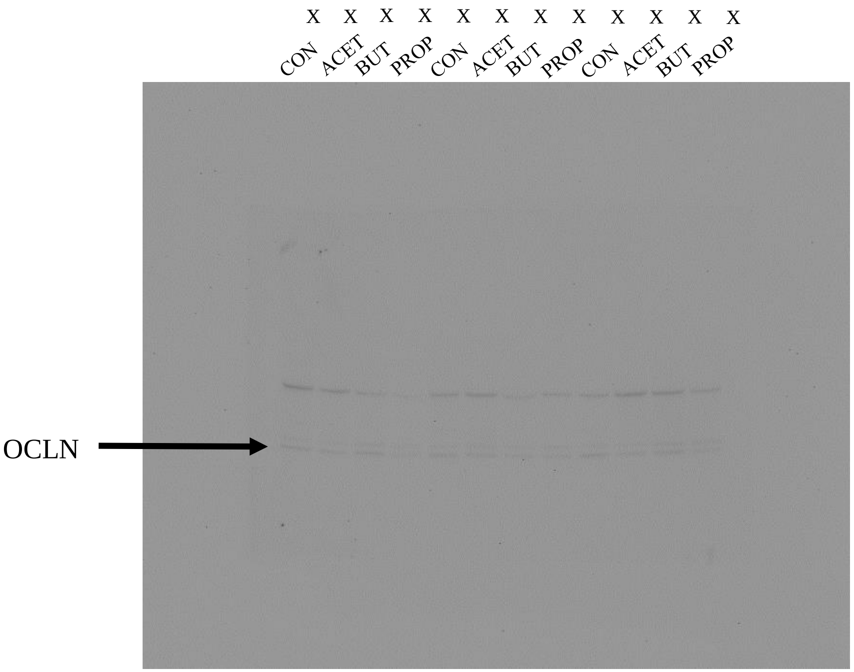

X – not used in final figure

Figure 7D

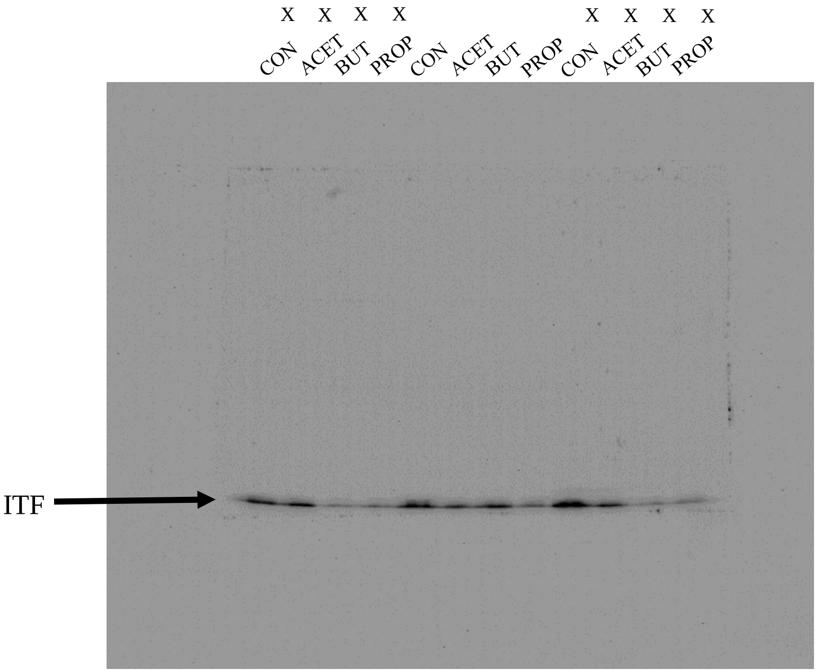

X – not used in final figure

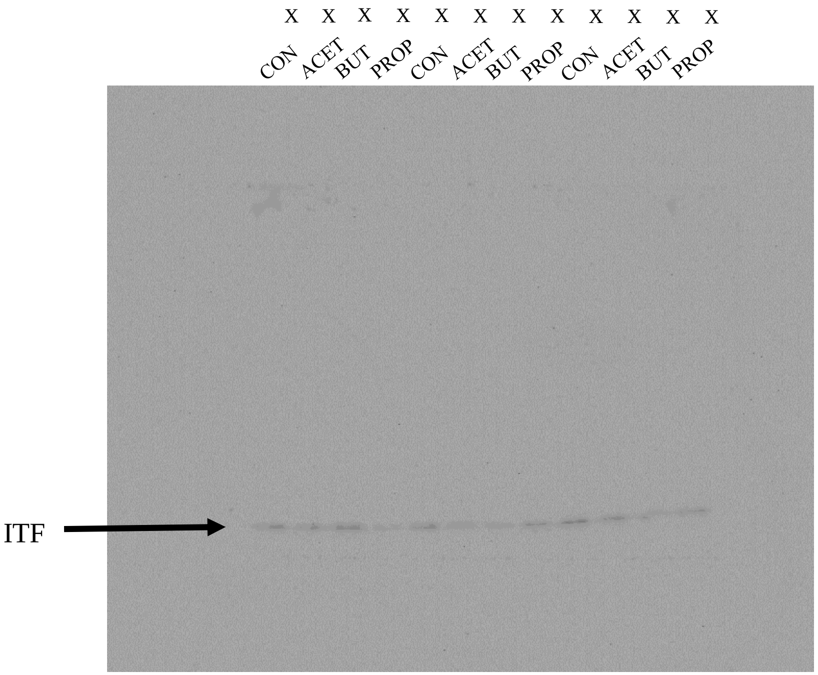

X – not used in final figure

Figure 7D

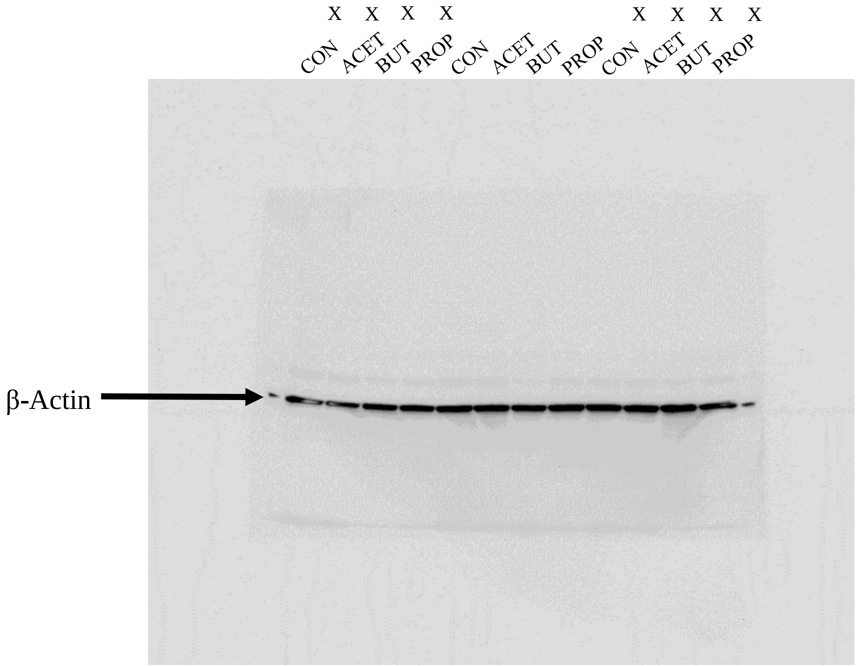

X – not used in final figure

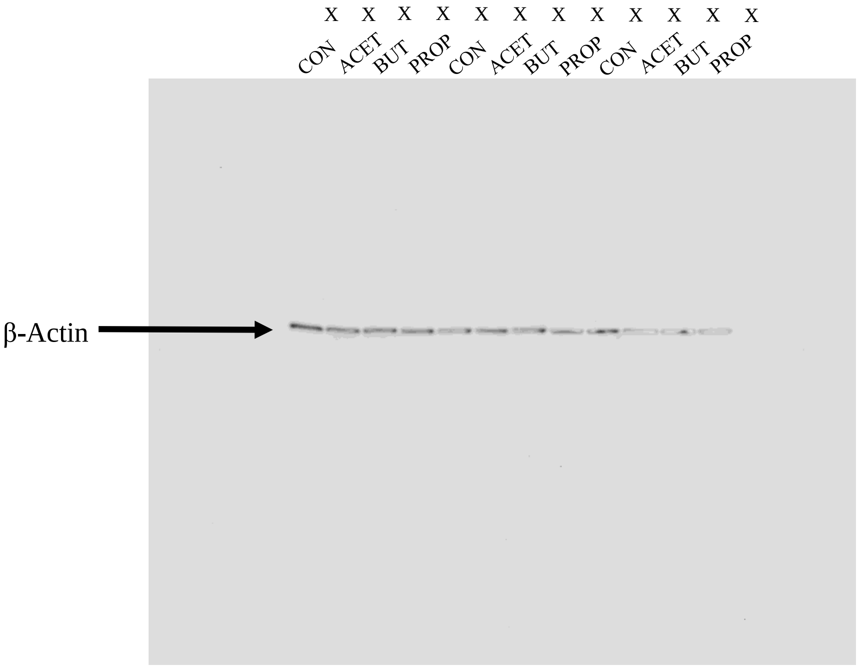

X – not used in final figure

Figure 7D
