# Supplementary material for: Intestinal enteroids recapitulate the effects of short-chain fatty acids on the intestinal epithelium
Source: PLoS One. 2020 Apr 2;15(4):e0230231. doi: 10.1371/journal.pone.0230231 (PMC7117711; doi:10.1371/journal.pone.0230231)
Supplement: S1 Table — (DOCX) [file pone.0230231.s004.docx]

| **Supplemental Table 1a**. Mouse Primer Sequences | | |
| --- | --- | --- |
| **Gene Name** | **Forward Primer 5’ to 3’** | **Reverse Primer 5’ to 3’** |
| β-actin (*Actb*) | TTG TTA CCA ACT GGG ACG ACA TGG | CTG GGG TGT TGA AGG TCT CAA ACA |
| Alkaline Phosphatase (*Alpi*) | GGC CAT CTA GGA CCG GAG A | TGT CCA CGT TGT ATG TCT TGG |
| Chromogranin A (*Chga*) | CGA TCC AGA AAG ATG ATG GTC | CGG AAG CCT CTG TCT TTC C |
| Claudin 3 (*Cldn3*) | ACC AAC TGC GTA CAA GAC GAG | CGG GCA CCA ACG GGT TAT AG |
| Doublecortin-like kinase 1 (*Dclk1*) | TCC ACC GGA ATT GAA CTC GG | GGG AGC GAA CAG TCT CAG A |
| β-Defensin 1 (*Defb1*) | AGC CAG GTG TTG GCA TTC TC | GCT TAT CTG GTT TAC AGG TTC CC |
| Leucine-rich repeat-containing G-protein coupled receptor 5 (*Lgr5*) | TGA GCG GGA CCT TGA AGA TT | AGG TGC TCA CAG GGC TTG AA |
| Lysozyme (*Lyz*) | ATG GCT ACC GTG GTG TCA AG | CGG TCT CCA CGG TTG TAG TT |
| Monocarboxylate transporter 1 (*Mct1*) | TGT ATG CTG GAG GTC CTA TCA G | CCA ATG CAC AAG TAA AGT TCC TG |
| Mucin 2 (*Muc2*) | CTT CTG TGC CAC CCT CGT | TTC GGG ATC TGG CTT CTT |
| Occludin (*Ocln*) | ATC CAC CTA TCA CTT CAG A | TAA TCT CCC ACC ATC CTC |
| Regenerating islet-derived protein 3 α (*Reg3a*) | GCA GAG TGG ACA ACT ACC AAG | GAC CAC GGT TGA CAG TAG AGG |
| Regenerating islet-derived protein 3 β (*Reg3b*) | ATG CTT CCC CGT ATA ACC ATC A | ACT TCA CCT TGC ACC TGA GAA |
| Regenerating islet-derived protein 3 γ (*Reg3g*) | ATG CTT CCC CGT ATA ACC ATC A | ACT TCA CCT TGC ACC TGA GAA |
| Tight junction protein 1 (*Tjp1*) | GCT TTA GCG AAC AGA AGG AGC | TTC ATT TTT CCG AGA CTT CAC CA |

| **Supplemental Table 1b.** Human Primer Sequences | | |
| --- | --- | --- |
| **Gene Name** | **Forward Primer 5’ to 3’** | **Reverse Primer 5’ to 3’** |
| β-actin (*Actb*) | CTC CTT AAT GTC ACG CAC GAT | CAT GTA CGT TGC TAT CCA GGC |
| Alkaline Phosphatase (*Alpi*) | TGA GGG TGT GGC TTA CCA G | GAT GGA CGT GTA GGC TTT GCT |
| Chromogranin A (*Chga*) | ACT CCG AGG AGA TGA ACG GA | CTT GGA GAG CGA GGT CTT GG |
| Claudin 3 (*Cldn3*) | AAC ACC ATT ATC CGG GAC TTC T | GCG GAG TAG ACG ACC TTG G |
| Doublecortin-like kinase 1 (*Dclk1*) | ACT TCG ACG AGC GGG ATA AG | GGG CCT CAA AAG ATC GGA ACC |
| β-Defensin 1 (*Defb1*) | ATG AGA ACT TCC TAC CTT CTG CT | TCT GTA ACA GGT GCC TTG AAT TT |
| Leucine-rich repeat-containing G-protein coupled receptor 5 (*Lgr5*) | TCA GTC AGC TGC TCC CGA AT | CGT TTC CCG CAA GAC GTA AC |
| Lysozyme (*Lyz*) | GGC CAA ATG GGA GAG TGG TTA | CCA GTA GCG GCT ATT GAT CTG AA |
| Monocarboxylate transporter 1 (*Mct1*) | GGT GGA GGT CCT ATC AGC AGT | CAG AAA GAA GCT GCA ATC AAG C |
| Mucin 2 (*Muc2*) | GGA GAT CAC CAA TGA CTG CGA | GAA TCG TTG TGG TCA CCC TTG |
| Occludin (*Ocln*) | ACA AGC GGT TTT ATC CAG AGT C | GTC ATC CAC AGG CGA AGT TAA T |
| Regenerating islet-derived protein 3 α (*Reg3a*) | TAC TCA TCG TCT GGA TTG G | ATC TTT CCA CCT CAG AAA TG |
| Regenerating islet-derived protein 3 β (*Reg3b*) | GGT GAG GAG CAT TAG TAA CAG C | CCA GGG TTT AAG ATG GTG GAG G |
| Regenerating islet-derived protein 3 γ (*Reg3g*) | GGT GAG GAG CAT TAG TAA CAG C | CCA GGG TTT AAG ATG GTG GAG G |
| Tight junction protein 1 (*Tjp1*) | CAA CAT ACA GTG ACG CTT CAC A | CAC TAT TGA CGT TTC CCC ACT C |
